# Differential Responses of Calcifying and Non-Calcifying Epibionts of a Brown Macroalga to Present-Day and Future Upwelling pCO_2_

**DOI:** 10.1371/journal.pone.0070455

**Published:** 2013-07-23

**Authors:** Vincent Saderne, Martin Wahl

**Affiliations:** GEOMAR, Helmholtz Center for Ocean Research in Kiel, Benthic Ecology Group, Kiel, Germany; Argonne National Laboratory, United States of America

## Abstract

Seaweeds are key species of the Baltic Sea benthic ecosystems. They are the substratum of numerous fouling epibionts like bryozoans and tubeworms. Several of these epibionts bear calcified structures and could be impacted by the high pCO_2_ events of the late summer upwellings in the Baltic nearshores. Those events are expected to increase in strength and duration with global change and ocean acidification. If calcifying epibionts are impacted by transient acidification as driven by upwelling events, their increasing prevalence could cause a shift of the fouling communities toward fleshy species. The aim of the present study was to test the sensitivity of selected seaweed macrofoulers to transient elevation of pCO_2_ in their natural microenvironment, *i.e*. the boundary layer covering the thallus surface of brown seaweeds. Fragments of the macroalga *Fucus serratus* bearing an epibiotic community composed of the calcifiers *Spirorbis spirorbis* (Annelida) and *Electra pilosa* (Bryozoa) and the non-calcifier *Alcyonidium hirsutum* (Bryozoa) were maintained for 30 days under three pCO_2_ conditions: natural 460±59 µatm, present-day upwelling1193±166 µatm and future upwelling 3150±446 µatm. Only the highest pCO_2_ caused a significant reduction of growth rates and settlement of *S. spirorbis* individuals. Additionally, *S.* *spirorbis* settled juveniles exhibited enhanced calcification of 40% during daylight hours compared to dark hours, possibly reflecting a day-night alternation of an acidification-modulating effect by algal photosynthesis as opposed to an acidification-enhancing effect of algal respiration. *E. pilosa* colonies showed significantly increased growth rates at intermediate pCO_2_ (1193 µatm) but no response to higher pCO_2_. No effect of acidification on *A. hirsutum* colonies growth rates was observed. The results suggest a remarkable resistance of the algal macro-epibionts to levels of acidification occurring at present day upwellings in the Baltic. Only extreme future upwelling conditions impacted the tubeworm *S.* *spirorbis*, but not the bryozoans.

## Introduction

Since the pre-industrial time, human activities led to an increase of the atmospheric pCO_2_ from 280 to 399 µatm [1]. CO_2_ is an acid gas and when dissolving in seawater, leads to the reduction of seawater pH. This phenomenon of ocean acidification has already caused a pH drop by 0.1 units since the year 1800. The most severe scenarios predict atmospheric pCO_2_ of up to of 1000 µatm to occur by the end of the 21^th^ century, decreasing the oceanic pH by a further 0.4 units [2]. This shift will increase the corrosiveness of seawater to calcium carbonate, a major component of most shells and skeletons of marine species. Consequently, in the course of ocean acidification, non-calcified organisms may outcompete calcifying organisms [3] as already observed between corals and seaweeds [4] or among seagrasses epiphytes in the Mediterranean Sea [5], [6].

The Baltic Sea is particularly prone to acidification due to its low salinity, alkalinity and temperature [7], [8]. This large scale/long term global signal may be overlain by a biological signal at a shorter and smaller scale, but of higher intensity. Indeed, in the nearshore macrophyte meadows of the Western Baltic, diurnal photosynthesis/respiration cycles of phytoplankton and benthic macrophytes drives day/night oscillations of pCO_2_ by 200 to 400 µatm, causing fluctuations by 1.5 to 2.5 of the saturation state for aragonite and calcite (Ω_arag_, Ω_calc_), the two isoforms of calcium carbonate [7], [9]. At the end of summer, the intensification of westerly winds together with the collapse of the thermocline leads to upwelling events of hypercapnic deep water masses to the nearshore habitats [10]. During such events, the mean pCO_2_ may reach 1600 µatm in the macrophyte stands, with an amplitude of diel variation up to 2180 µatm [9]. Under such circumstances, the nearshore habitats may remain undersaturated for calcite and aragonite for periods of days to weeks.

As for the open ocean, the pCO_2_ in the Baltic Sea is predicted to increase during the 21^st^ century, but the value of that increase is unknown [11]. This uncertainty for the Baltic sea is caused by the concomitant shift of several other parameters: increase of mean sea surface temperature by 2 to 3.5°C, increase of the riverine input of dissolved organic carbon, strengthening of westerly wind (affecting upwelling and exchanges with the North Sea) and decrease of sea surface salinity [12], [13]. This multi-factorial change is expected to increase the duration, frequency and intensity of the upwellings events [14]. Melzner *et al*. (2012) [14] have shown a relationship between hypoxia and elevated pCO_2_ in the upwelling deep water of the Baltic. According to their model, the pCO_2_ recorded in 2011 by Saderne *et al*. (2013) [9] in Baltic macrophyte meadows would correspond to concentrations of O_2_ in deep water of approx. 100 µM (instead of a theoretical saturated O_2_ concentration of approx. 290 µM at salinity 20 and temperature 13°C [9], [15]). For this level of hypoxia and considering a doubling of atmospheric pCO_2_ during the 21^st^ century [2], the model of Melzner *et al*. (2012) [14] predicts the pCO_2_ to reach 3000 µatm in the macrophyte meadows during the end of summer upwelling in 2100.

The brown alga *Fucus serratus* is a common and, locally, dominant species in the shallow (<3 m) macrophyte meadows in the Western Baltic, providing valuable ecosystem services such as primary production, the provision of spawning and nursery areas, food for herbivores and substratum for micro- and macrofoulers [16], [17]. In total, 164 macro- and micro-faunal species have been inventoried as associated with *F. serratus* in the Baltic [18]. Most prevalent sessile epibiotic species inventoried are the calcifying tubeworm *Spirorbis spirorbis*, the calcifying bryozoan *Electra pilosa* and the non-calcifying bryozoan *Alcyonidium hirsutum* [18]. As most epibionts, these species play an important role in the ecology of their host. *S. spirorbis* can overgrow fucoid blades, presumably causing shading and an increase in weight and brittleness [19]. Similarly, shading by *E. pilosa* and *A. hirsutum* can reduce the photosynthetic activity of *F. serratus* by up to 85% [20] and their physical presence could contribute to the degeneration of the underlying thallus as demonstrated on kelps [21], [22]. In kelp forests, the overgrowth by bryozoans can lead to extensive defoliation affecting the entire ecosystem [23], [24]. On the other hand, bryozoans are directly supplying the host algae with ammonium [25] and dissolved inorganic carbon (DIC) [26] and are a food source for grazers such as nudibranchs and urchins [27]–[29].

The epibiotic species spend at least the initial part of their life cycle within the diffusive boundary layer (DBL) surrounding the algal thallus. This layer typically is 50 µm to 2 mm thick depending on ambient flow velocity [30], [31]. It is characterized by the slow diffusion of molecules, creating steep concentration gradients of compounds produced or consumed by the alga and its micro- and macro-epibionts [30]–[33]. Due to the net uptake of CO_2_ at daytime and the net release of CO_2_ at night, pH within the DBL of *Fucus vesiculosus* can vary between 8 in the dark and 9.2 at 785 µE light [34]. The biotic signal is apparently skewed towards lower pH *i.e.* algal photosynthesis increases pH more than algal respiration decreases it (*e.g.* [34]). Consequently, we would expect the microhabitat on the macroalga thallus to shelter sessile calcifiers from seawater corrosiveness, especially during daytime [31].

In the present study, we tested the hypothesis whether present-day and future upwelling pCO_2_ (“transient acidification”) (i) impacts epibiotic species and (ii) disadvantages the calcifying sessile fauna of *F. serratus* over the non-calcifying one. The calcifying tubeworm *S. spirorbis* and two bryozoan species, the calcifying cheilostome *E. pilosa* and the keratinous ctenostome *A. hirsutum* were investigated regarding their growth and settlement (*S. spirorbis* only) under three pCO_2_ conditions during a 30 day incubation. The two higher pCO_2_ treatments were meant to simulate a prolonged upwelling event of variable intensity. Treatments were unmodified pCO_2_ (460 µatm), typical present-day upwelling pCO_2_ (1200 µatm) and upwelling pCO_2_ expected by 2100 (3150 µatm). We also tested at the same pCO_2_ light versus dark growth of epibiotic *S. spirorbis* settled juveniles as a first indicator of a potential protection of calcification in the DBL by the host algae metabolism.

## Materials and Methods

### Collection of Macroalgae and Epibionts

*Fucus serratus* individuals bearing epibiotic communities composed of *S. spirorbis*, *E. pilosa* and *A. hirsutum* were collected in less than 2 m depth in Eckernförde Bay (Western Baltic Sea, Germany, 54°27′ N, 9°53′ E) on the 1^st^ of February 2011, carried in coolers to the lab and stored in one aquarium (100 L) for two days under the conditions of pCO_2_ (460 µatm), light (300 µE), salinity (17) and temperature (16°C) of the control treatment of the future incubation. No specific permits were required for the study, the location is not privately-owned or protected in any way and the study did not involve endangered or protected species.

Bryozoans are colonial filter feeders (as *e.g*. corals), composed of modules called zooids expending by asexual budding. Bryozoans are protected by box-like exoskeletons of structure differing among taxa. In ctenostome bryozoans such as *A. hirsutum*, the skeleton is a periostracum composed of a thin membrane covering a main periostracal layer, both made of keratin and muco-polysaccharides [35]. In cheilostomes such as *E. pilosa*, the above described periostracum is reinforced on the inside by a layer of calcium carbonate [35]. The biomineralization pathway is still unexplored [36].

Each zooid is connected to its neighbors by channels through the skeleton, allowing the allocation of resources to the budding edge of the colony. Within a colony, zooids are deciduous units. Each filter-feeding zooid can only ingest a determined amount of food before the accumulation of waste within the stomach epithelium leads to the zooid degeneration [37], [38]. The zooid is therefore replaced by a new one within the same skeletal box.

*S. spirorbis* is a filter feeding tubeworm of maximum 5 mm length protected by a spiral calcified tube. The tube is secreted by glands located under a collar between the head and the thorax [39]. According to Nott and Parkes (1975) [39] the crystals of calcium carbonates are formed in the lumen of the gland from a calcium rich mucous secretion and carbonate and bicarbonate ions originating from seawater. Embryos are brooded within the calcareous tube in an egg bag attached to the parental body [40]. The release of swimming non-feeding larvae is triggered by favourable environmental factors (rising tide, especially of spring type) [41]. The settlement and metamorphosis into juveniles occurs within 1 to 3 hours [41].

### Preparation of the Material

**Figure 1:**
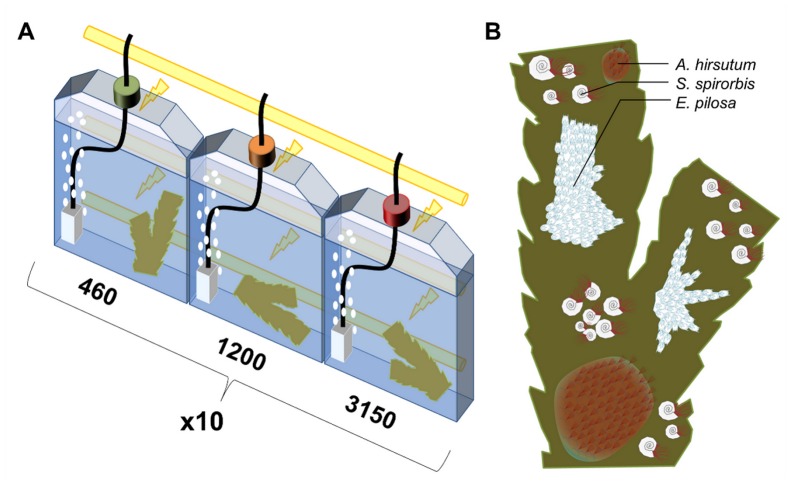
Experimental design. (**A**) Schematic representation of the incubation system. (**B**) Schematic representation of one experimental *F. serratus* section bearing the three epibionts, both sides of the thallus were bearing epibionts.

Two days after sampling, equivalently fouled thallus sections of *F. serratus* were cut to an average (± SD) weight and area of 1±0.02 g and 24±5 cm^2^, reciprocally. Sections were taken at the center of the thallus (Fig. 1). We took care not to hurt the epibionts in the process. Each thallus section carried on average (± SD) 26.8±5.3 *S. spirorbis* individuals, 6.2±1.9 colonies of *E. pilosa* and 1.9±0.6 colonies of *A. hirsutum*, distributed on both sides of the thallus. The mean (± SD) colonies area was 2.3±3.6 cm^2^ for *E. pilosa* and 3.5±3.8 cm^2^ for *A. hirsutum*. The fluorescent dye calcein was added for five days at 50 mg L^−1^ to the aquarium (in the conditions of incubation stated in the previous sub-section).

The incorporation of the dye into the newly grown tubes and bryozoan skeletons marked the starting point for calcification under the experimental treatment levels [42]–[44]. Calcein is a very low toxicity dye often used in biomineralization studies, as on the bryozoan *Adeonellopsis sp* [45]. The innocuty of calcein at concentrations inferior to 100 mg L^−1^ has been demonstrated on bivalves [46] and juvenile fish [47].

During the 5 day staining period, the animals were fed *ad libitum* with the microalgae *Rhodomonas sp.* to ensure good growth rates [48], [49] and intense incorporation of the stain. The expansion of the tubes and colonies during the calcein staining attested for the absence of stress in the animals linked to the staining or, generally, to the maintenance conditions.

### Experimental Design

A schematic representation of the experimental design is given in Fig. 1. Each *F. serratus* section bearing the three epibiont species was individually incubated in a 600 mL culture flask (11 cm×3.5 cm×14.5 cm; Sarstedt, Germany). Ten replicate flasks were assigned to each pCO_2_ treatment. Flasks were constantly aerated with modified ambient air containing either 460 µatm pCO_2_ (ambient), 1200 µatm pCO_2_ or 3000 µatm pCO_2_ (Linde gas and HTK Hamburg, Germany) (see [50] for details). The vigorous gas bubbling assured a continuous convective movement of water and a homogeneous repartition of microalgae in the flasks as in Riisgård and Goldson (1997) [51]. The algae sections and the epibionts were neither in contact with the bubbling stone nor with the bubbles but were confined to the opposite side of the experimental flasks by the convective flow.

Temperature was maintained at 16°C and light was provided by three Biolux neon tubes (Osram, Germany), delivering a total of 300 µE under a 12/12 h day/night cycle. The seawater of the flasks was replaced every third day with water pre-adjusted to the same pCO_2_ and enriched with *Rhodomonas sp*. to a final concentration of 10,000 cells mL^−1^, corresponding to the phytoplankton concentrations observed in August – September in the inner bays of the Western Baltic [52].

Natural Baltic seawater from the Kiel Bight (Western Baltic Sea, Germany, 54° 19′ N, 10° 08′ E) was used in the experiment after storage in a 300 L tank aerated with ambient air and sterilized by a Microfloat 1 floating UV lamp (Aqua Concept Karlsruhe, Germany). 24 h before use, the seawater was equilibrated with the three experimental pCO_2_ in three separate tanks.

The duration of the incubation was 30 days, simulating the duration of an extended present-day upwelling period and of a possibly average duration of future upwelling events. At the end of the incubation period, the algal fragments were conserved in borax-formaldehyde-seawater solutions suitable for preservation of calcareous structures (see [53] for the detailed recipe) prior to the measurement of growth rates.

### Measurement of Relative Growth Rates (RGR) of Bryozoans

We used relative growth rates to compare the growth rates of colonies of different starting sizes. Colonies were photographed by overlapping fields of vision under an epifluorescence microscope (Axio Scope A1, Carl Zeiss, Germany) at the end of the experiment. The partial pictures were reassembled into complete colony pictures. The initial colony surface (SI) (pre-staining) and the final total colony surface (SF) (after 30 days incubation) were measured by image analysis (ImageJ, U. S. National Institutes of Health). In each replicate flask, SI and SF of all the colonies were summed for each species so that in each flask (replicate) the relative growth rate in percent (RGR) was:
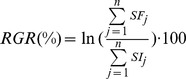



With *n* the number of colonies in one flask. The logarithmic growth pattern of bryozoan colonies has been assessed by [48]. *A. hirsutum* colonies (non-stainable), were photographed prior and after incubation. The areas were measured and the RGR calculated as for *E. pilosa*.

### Measurement of Growth and Settlement of *S. spirorbis*

**Figure 2:**
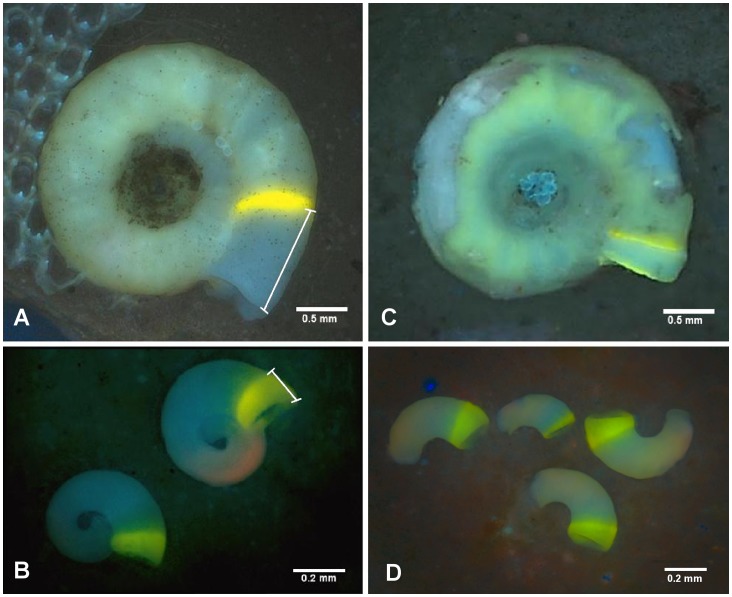
Illustrative examples. (**A, C**) Adult *S. spirorbis* tubes after 30 days of incubation at 460 µatm and 3150 µatm pCO_2_ respectively. (**B, D**) Juveniles *S. spirorbis* tubes after 30 days of incubation at 460 µatm and 3150 µatm pCO_2_. Note the conspicuous dissolution of the shell in (**C**) and the disappearance of part of the tube in (**D**). All photos were taken under epifluorescence microscope, yellow/green: calcein staining. The white lines show the considered distances for the growth measurements of *S. spirorbis* tubes.

For *S. spirorbis*, the absolute tube growth in mm was estimated as the length of the external arc of the coil comprised between the staining front and the terminal tube edge around the opening (see Fig. 2 A). In each flask, the new tube length increment of all worms was averaged as the flask represents one replicate within a treatment level. *S. spirorbis* settlement was quantified for each flask as the number of juveniles found on algae sections (their favorite substratum) at the end of the experiment divided by the number of adults. No juveniles were present on the thallus at the onset of the experiment.

### Growth of *S. spirorbis* Juveniles under Light and Dark Conditions

To test the effect of photosynthetic activity and pCO_2_ on the growth of juvenile worms, calcein was added to all the flasks (final concentration: 20 mg L^−1^) during the last 24 hours of the experiment. Subsequently, half of the flasks were kept in light (with host algal photosynthesis), the other half was darkened by wrapping in aluminum foil (without host algal photosynthesis). Growth was measured as the length of the stained newly formed tube (see Fig. 2 B).

### Seawater Chemistry

Seawater processing for carbonate system measurements was made according to the standard operating procedure (SOP) 1 of Dickson and Sabine (2007) [54]. Three random flasks in each treatment and the three mixing tanks were sampled prior to each water exchange. The temperature of the flasks and the tanks were measured with 0.01°C precision for future in-situ pH recalculation (see below). Samples were analyzed in the laboratory for DIC and pH_T_. DIC was measured with an AIRICA (Marianda, Germany), the measurement principle is based on the infrared measurement of CO_2(g)_ purged out of an acidified sample. The system was calibrated on every measurement day with Certified Reference Material (CRM) (Andrew Dickson, Scripps Institution of Oceanography). The pH on total scale (pH_T_) was measured as follows. Seawater TRIS pH buffers at salinity 15 were made according to the SOP 6a [54]. A combined reference/measurement electrode Metrohm Ecotrode (Metrohm, Switzerland) was used together with a pH-meter/conductimeter Mettler-Toledo SG 7/8 (Mettler-Toledo, Switzerland). For calibration, the TRIS buffer was immersed in a thermostatic bath and the voltage (±0.1 mV) of the pH electrode was measured. The temperature of the buffer was modulated on a range of 1.5°C. The temperature corresponding to every mV change was recorded with >0.01°C accuracy with a Fluke 5658 reference thermometer equipped with a 5608 platinum resistance sensor (Fluke, USA). The procedure was repeated by increasing and decreasing the temperature to get an average voltage (mV) *versus* T (°C) reference curve for the electrode in the buffer. The fitting of the electrode voltage to the curve was assessed on every measurement day to check for the natural depolarization of the electrode. This calibration protocol was repeated at least once a week. The voltage and temperature of the sample was measured at the temperature corresponding to the calibration range. The sample voltage, sample temperature and the theoretical TRIS buffer voltage (corresponding through the calibration curve to the sample temperature) were computed through the equation of the SOP 6a to obtain the laboratory pH on total scale of the sample. The temperature of the sample measured the climate room at the time of sampling was used to recalculate the in-situ pH. Preparative work conducted on CRM together with buffers at salinity 35 demonstrated an accuracy of this method of 0.003 to 0.005 pH units. Salinity of the samples was measured to an accuracy of 0.01 with a Mettler Toledo Inlab 738 conductivity probe after calibration at 25°C with KCl 0.1 mol L^−1^ (Fischer Scientific, USA). The overall in situ-pH and carbonate system was recalculated with the R package Seacarb [55] using first and second carbonate system dissociation constants for estuarine systems from Millero (2010) [56] and the dissociations constants of HF and HSO_4_^−^ of Perez and Fraga (1987) [57] and Dickson (1990) [58].

### Statistical Analysis

Statistical analyses were conducted with Statistica 7 (Statsoft, USA.). Treatments were compared using one way ANOVAs and Tukey’s HSD tests. Assumptions of normality and homoscedasticity were tested with Shapiro-Wilk’s and Levene’s tests. To achieve normality, data for growth rates of *E. pilosa* and *A. hirsutum* were transformed by the natural logarithm.

## Results

### Chemistry

A summary of the variables of the carbonate system measured in the flasks during the 30 days duration of the experiment derived from pH_T_ and DIC is presented in Table 1. Average (± SD) pCO_2_ treatment levels were 460±59 µatm, 1193±166 µatm and 3150±445 µatm. Saturation states (mean ± SD) below one were found for aragonite only (0.82±0.14) in the bulk water of the 1200 µatm treatment and for both isomorphs (Ω_calc_ 0.66±0.14, Ω_arag_ 0.40±0.09), was found in the 3150 µatm pCO_2_.

**Table 1:** Seawater carbonate system in the experimental flasks.

Treatment	T (°C)	Sal	pH_T_	pCO_2_ (µatm)	DIC (µmol kg^−1^)	A_T_ (µmol kg^−1^)	Ω_calc_	Ω_arag_
**460 µatm**	16.1±0.7	16.7±0.4	8.105±0.031	450±59	1966±40	2089±52	3.0±0.3	1.8±0.2
**1200 µatm**	15.7±0.5	16.7±0.4	7.726±0.076	1193±166	2115±62	2135±71	1.4±0.2	0.8±0.1
**3150 µatm**	15.9±0.8	16.8±0.4	7.334±0.079	3150±445	2306±251	2298±57	0.7±0.1	0.4±0.1

Data derived from DIC and pH_T_, data are the means ± SD of three flasks sampled every three days before water exchange, along the 30 days duration of the experiment. (n = 10).

### Spirorbis spirorbis

**Figure 3:**
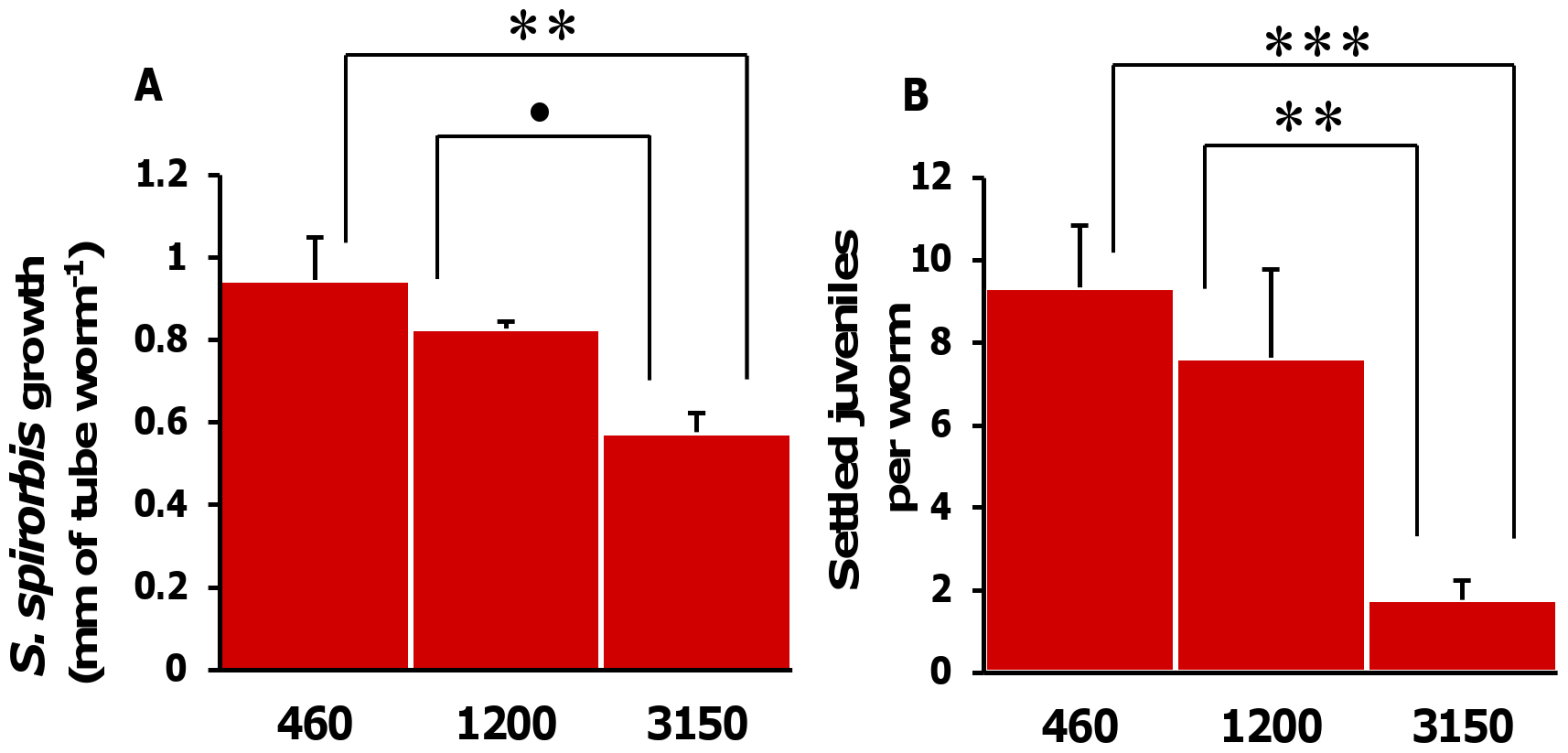
Growth and reproduction of *S. spirorbis*. (**A**) Growth of the tubes of *S. spirorbis* during the 30 days of incubations at 460 µatm, 1200 µatm and 3150 µatm pCO_2_, data are the mean ± SE of newly formed tubes sections in mm per worm per flask. (**B**) Settlement of *S. spirorbis* during the 30 days of incubations at 460 µatm, 1200 µatm and 3150 µatm pCO_2_, data are the mean ± SE of the number of juveniles settled per adult between flasks. One-way ANOVAs, n = 10, statistical significance: ***: p≤0.001, **: p≤0.01, *: p≤0.05, •: p<0.1.

**Figure 4:**
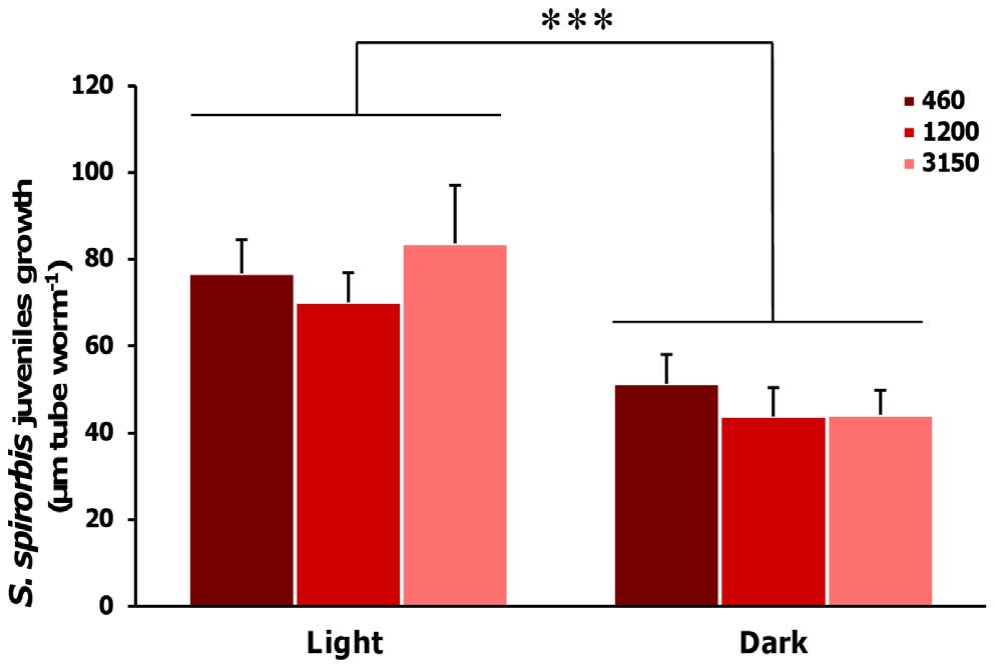
Growth of the juveniles of *S. spirorbis*. Growth in µm of the tubes of the juveniles of *S. spirorbis* during 24 h in light and dark condition exposed to pCO_2_ of 460 µatm, 1200 µatm and 3150 µatm. Data are the mean ± SE newly formed tubes sections in µm per worm per flask Two-way ANOVA, n = 5, statistical significance: ***: p≤0.001.

The growth of *S. spirorbis* tubes was significantly affected by pCO_2_ (one way ANOVA, Df = 2, F = 5.84, p 0.008, Fig. 3 A). Compared to the 460 µatm, reductions of growth rates by approx. 40% were found at 3150 µatm (Tukey’s HSD, p = 0.007) and by approx. 25% at 1200 µatm (marginally significant, Tukey’s HSD, p = 0.075). While no signs of shell injuries were visible at 460 µatm and 1200 µatm, all *S. spirorbis* tubes exhibited substantial shell dissolution marks at 3150 µatm (see Fig. 2 C). This loss of integrity of the outer spiral of the tube exposed the worm bodies and the embryo bags to the external seawater. Settlement of *S. spirorbis* was significantly affected by pCO_2_ (one way ANOVA, Df = 2, F = 12.98, p<0.001, Fig. 3 B). The number of settled juveniles per adult was significantly reduced by approx. 75% at 3150 compared to 1200 µatm (Tukey’s HSD, p = 0,003) and by approx. 85% compared to the control (Tukey’s HSD, p<0.001). Juvenile tube growth was significantly higher by about 40% in light as compared to dark incubation (factorial ANOVA, Df = 1, F = 17.6, p<0.001; Fig. 4). The pCO_2_ did not significantly influenced the juvenile growth rates (factorial ANOVA, Df = 2, F = 0.44, p = 0.64) but important damages on the early tubes were observed in the 3150 µatm treatment (see Fig. 2 D).

### Bryozoans

**Figure 5:**
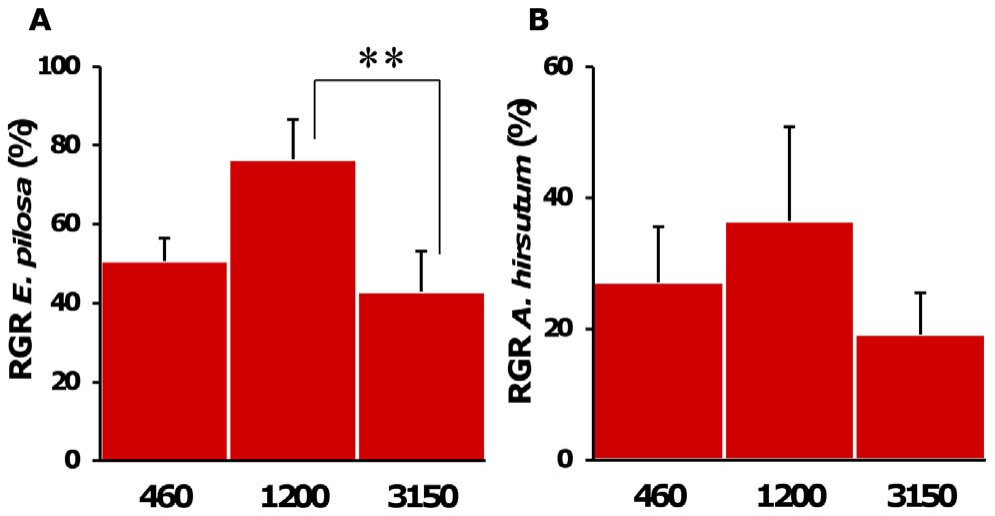
Growth of the bryozoan. Relative growth rate (RGR) of (**A**) *E. pilosa*, (**B**) *A. hirsutum* colonies during the 30 days of incubations at 460 µatm, 1200 µatm and 3150 µatm pCO_2_. Data are the mean ± SE of the growth rates in % in n = 10 flasks. One-way ANOVAs, statistical significance: **: p≤0.01.

The growth rate of *E. pilosa* was significantly affected by pCO_2_ (one way ANOVA, Df = 2, F = 5.04, p = 0.014). Growth rates were enhanced at 1200 µatm by approx. 35% compared to 3150 µatm and by approx. 45% compared to the control. However, only the difference between the 3150 µatm treatment and the 1200 µatm was significant (Tukey’s HSD, p = 0.01) (Fig. 5 A). In the highest pCO_2_ treatment the skeleton of uninhabited zooids, but not of live zooids, dissolved completely resulting in a “ghost” organic matrix of the exact same shape. The growth rates of *A. hirsutum* were similar under all pCO_2_ levels (ANOVA, Df = 2, F = 0.4, p = 0.64; Fig. 5 B).

## Discussion

None of our experimental species was negatively impacted in growth rates or settlement (investigated only for the polychaete) under present-day upwelling pCO_2_ conditions (1200 µatm treatment). Instead, bryozoans tended to grow better at this pCO_2_. At the pCO_2_ level expected in 2100 upwelling events (3150 µatm treatment), *S. spirobis* showed a severe reduction of growth and settlement together with important tube dissolution while both bryozoans, calcifying and non-, were unaffected. Therefore, we cannot validate the hypothesis of a *general* competitive advantage of non-calcifying epibionts over calcifying ones at elevated pCO_2_.

We observed as a trend a quadratic relation between bryozoans growth and increasing pCO_2_ with best growth at intermediate levels of pCO_2_. Similar response patterns were described by Ries et *al.*, 2009 [59] for several calcifiers exposed to lowered pCO_2_. In contrast, a tendency toward a more linear reduction of growth rates with increasing pCO_2_ was found in the calcifying bryozoan *Celleporella hyalina* (max. pCO_2_ tested: 4400 µatm, [60]) and *Celleporaria nodulosa* (max. pCO_2_ tested: 1200 µatm, [61]) albeit with remarkable intraspecific variability. This (presumably) genetic variability of sensitivity hints at a substantial potential of adaptation of these bryozoan species to elevated pCO_2_ [60], [61].

The difference in sensitivity to acidification between the two calcifiers *S. spirorbis* and *E. pilosa* and the lack of difference between the two bryozoans, calcifying or not, could be due to differences in physiology, crystallization pathways, shell mineralogy and/or structuration. Most of these are unknown for the three species investigated, however Bornhold and Milliman (1973) [62], in what remains the only study documenting the mineralogy of *S. spirorbis* (as *S. borealis*), reported that their tubes are calcitic with 10 to 14% of aragonite and include 0 to 10% of MgCO_3_ depending on location and possibly on environmental factors such as temperature. The skeleton of *E. pilosa* is exclusively calcitic, including 4 to 8% MgCO_3_ [63]–[65] and may, therefore, be less prone to dissolution than the worm tubes. This could explain some of the observed sensitivity differences between the two calcifiers. Furthermore, Nott and Parkes (1975) [39] suggested for *S. spirorbis* that the crystallization process directly depends on the uptake of DIC from seawater. Therefore, the reduction of the worm growth observed at elevated pCO_2_ could also be explained by the reduction of the carbonate ion fraction of the DIC.

To our knowledge, the crystallization pathway in the biomineralization of cheilostome bryozoans has not yet been described [36]. According to Weiner and Addadi (2011) [66], the fact that their skeleton can be labeled by calcein, a molecule that cannot pass through membranes, suggests that the deposited calcium originates from direct endocytosis of seawater. We cannot deduce anything concerning the origin of the carbonate involved (external or metabolic). For *E. pilosa*, the calcium carbonate is deposited beneath an organic periostracum made of muco-polysaccharides, proteins and chitin [35], protecting the carbonated skeleton from corrosive seawater. In contrast, the calcium carbonate of the spirorbid tube is in direct contact with seawater [67]. The protection or not of calcified structures by external organic layers and periostracum is recognized as a major feature explaining the differences of sensitivity to corrosive water between calcifier taxa [59], [68]. The organisms with protected shells or skeleton (*e.g.* corals and mussels) are more resilient to seawater corrosiveness than organisms with bare calcified structures (*e.g.* limpets) [59]–[68]. That feature could partially explain why we observed for *S. spirorbis* a reduction of growth for adults and important shell damages for adults and juveniles at 3150 µatm, when Ω_calc_ <1, but no effects for *E. pilosa*. For this latter specie, only the dead central parts of the colonies were decalcify, similarly to what was observed for the bryozoan *Myriapora truncata* at the CO_2_ vents of Ischia (Italy) [69]. This suggests the importance of living processes in bryozoans for the maintenance of the calcified structure. Our results for *S. Spirorbis* and *E. pilosa* are coherent with the observations made in Ischia for *Electridae* and *Spirorbidae* epibionts on the seagrass *Posidonia oceanica*. Cigliano *et al*. (2010) [70] found *Spirorbis marioni* only at stations of normal atmospheric pCO_2_ and Ω_calc_ >5.5. In our study, *S. spirorbis* was impacted for growth and settlement only at the highest pCO_2_ treatment with Ω_calc_ <1. As in the present study, Martin *et al*. (2008) [6] did not find any correlation between lowered pH and Ωs and the abundance of bryozoans, including *Electra posidoniae*.

In bryozoans, the fact that the calcified structure is just one element of a composite skeletal assemblage could explain the similarity of results between *E. pilosa* and *A. hirsutum*. To our knowledge, the skeleton of the genus *Alcyonidium* is mostly composed of chitin but its structure (architecture) has never been described in detail [71]. Instead, Tavener-Smith and Williams (1972) [35] described the composition and structure of *Bowerbankia sp.*, another ctenostome bryozoan. Compared to cheilostomes, ctenostome bryozoans have a thicker periostracum reinforced in chitin and lack any calcium carbonate layer. Cheilostomes evolved calcification from a soft-bodied ctenostome-type ancestor (of which *Alcyonidiidae* is the closest living related taxa [72]). Both clades share similar skeleton, differing only by the presence of the calcium carbonate layer [35], [36], [72]. The similarity of response of *E. pilosa* and *A. hirsutum* observed in the present study suggests that acidification impacts other physiological pathways than calcification common to both species with, as result, an increase of growth rates in the intermediate CO_2_ treatment. Such enhancement have been observed in some echinoderms [73], [74], but could be at the cost of energetic tradeoffs detrimental to other key functions like reproduction.

To our knowledge, the present article is the first presenting measures of the growth rates of *S. spirorbis*, adults and juveniles, and of *A. hirsutum*. In contrast, growth rates for *E. pilosa* have been assessed in several articles, under different food and flow regimes (reviewed by [48]–[75]). The growth rates in our experiment for *E. pilosa* are approximately ten times lower than observed in other laboratory experiments conducted with comparable concentrations of food. However, those studies have been conducted on isolated colonies in absence of competitors for food. In our study, each *E. pilosa* colony is in competition with conspecific colonies, with colonies of *A. hirsutum* and with *S. spirorbis* individuals. Consequently, the available food quantity for each colony might have been reduced close to the minimum threshold for growth of 500 cell mL^−1^ identified for *E. pilosa* [75].

The imposed water motion in our experimental flasks could have impaired the food uptake by the bryozoans, contributing to the reduced growth rates observed for *E. pilosa* [76]. The question of the influence of current velocity on bryozoans food uptake and growth is controversial [48], [76]. Hermansen *et al*. (2001) [48] stated that while very slow flow could impair supply to the polyps, high current velocities do not limit *E. pilosa* growth rates. In contrast, earlier studies by Okamura (1988, 1992) [77], [78] shown that strong current reduces feeding rates of isolated *E. pilosa* colonies but enhances feeding rates of both *A. hirsutum* and *E. pilosa* colonies when they occur contiguously on the same algal thallus. This facilitation between the two bryozoans would be due to micro-turbulences created by *A. hirsutum* colonies, slowing and diverting the flow at the vicinity of *E. pilosa* colonies [77].

Finally, we cannot exclude the slow growth rates to be the consequence of stresses relative to the shortness of the acclimation period (two days) and/or to the conditions of incubation (*e.g*. temperature, light). Regarding vigorous bubbling and short acclimation time, our study conditions resemble those of Okamura (1988) [77], Okamura (1992) [78], Mercado *et al*. (1998) [79] and Riisgård and Goldson (1997) [51] on similar or the same organisms. The absence of acclimation to elevated pCO_2_ through a stepwise acidification was deliberate and aimed at mimicking the sudden occurrence of an upwelling event [9].

Growth rates in *E. pilosa* were approx. 50% higher than in *A. hirsutum*. To our knowledge, no studies investigated the growth rates of both species in competition on a same algal thallus. Nonetheless, our results are in accordance with the known strategy for substrate occupation of both species. On *F. serratus*, *Alcyonidiidae* are end-of-succession species, slowly overgrowing all other epibionts until dominating the entire algal fronds [80]–[83]. In contrast, *E. pilosa* is a smaller, early-succession specie, mostly relying on elevated growth rates to escape overgrowth by superior bryozoan competitors [80]–[83].

A reduction of *S. spirorbis* settlement of about 85% relative to the control was found at the highest pCO_2_. In this species, the embryos are bred within the parental tube for a period of 10 to 25 days until the emission of a fully formed larvae settling within few hours [41], [84]. The reduction in settlement could be the result of adverse conditions during fertilization, cleavage (embryogenesis), planktonic larva, settlement or metamorphosis [85]. Exchange of sperm might or might not have occurred during the experiment. Some sperm stored over winter in a specific organ of the head could have been used to fertilize the eggs [86], [87]. Likewise, some embryos in dormancy at the beginning of the experiment could have reactivated their development. A more probable explanation for our results would be a delaying or interruption of the embryogenesis due to the exposure of the embryo bags to external acidified seawater after dissolution of the parental tubes. Such impacts during embryogenesis have been observed for gastropods, bivalves and echinoderms [88]–[90]. While the planktonic phase is considered a sensitive life stage (*e.g.* [85], [91]), the short duration of the pelagic phase in *S. spirorbis* (a few minutes to 3 h) and the non-calcifying nature of the larvae should reduce the likeliness of impact of acidified seawater. *Spirorbis spirorbis* specifically settles on brown algae and this preference is most likely chemically mediated [92]–[94]. The sensitivity to cues could be affected by seawater pH, making the larvae incapable of recognizing their hosts as found in clown fish larvae (*Amphiprion percula*), becoming “blind” to their host anemones cues under high pCO_2_ [95].

Our study did not considered species in isolation, but in close association with their host alga, reflecting the intention to assess the response of an ecologically relevant association to upwelling-like acidification events. Our results suggest that the host algae photosynthesis could modulate the calcification of the smallest encrusting organisms inhabiting the algal DBL. The thickness of the DBL is mostly depending on the flow velocity, ranging from almost 2 mm in stagnant water and exponentially decreasing to a threshold of 50 to 150 µm at flow velocity above 10 cm s^−1^ [30]–[32]. In the experimental flasks a vigorous and convective water motion was ensured by the continuous gas bubbling which, however, was not measured. The water movement served to ensure homogeneous conditions throughout the water column and to warrant exchanges of nutrients and gases at the thallus surface. While water movement *per se* was strong the shear velocity at the thallus surface was presumably moderate. We could expect therefore the thickness of the DBL to be of some intermediate size, maybe between 100 and 500 µm, such as *S. spirorbis* juveniles only might fully experience its effects. The conditions within algal boundary layer is strongly influenced by the physiological activity of the host algae and the micro- and macro-epibiont species living in it (*e.g*. [33] and references therein). For instance, during alternating cycles of algal respiration (“night”) and photosynthesis (“day”), oxygen concentration and pH at 100 µm above the thallus surface can vary between 200 and 750 µmol O_2_ L^−1^ and between 8.0 and 9.2 pH units, respectively (data extracted from plots in [34]). Thus, although this has never been investigated to date, one would expect that ocean acidification effects would be dampened in the DBL during daytime at least. Supporting this, we found a 40% enhancement of growth rates of juveniles under light compared to dark. In contrast, we did not find an antagonistic interaction of darkness (algal respiration) and elevated pCO_2_ on the juvenile growth rates. Therefore, we cannot exclude that the day/night rhythm of calcification observed could be due to inherent activity patterns of the worm or to stresses engendered by *e.g*. the variations of O_2_ more than by daily variations of Ω in the DBL.

In our experiment we found that the epibionts of *F. serratus* tolerate a transient rise of pCO_2_ similar to present-day upwelling events of the Western Baltic. We hypothesize that this tolerance consists of a periodic relaxation of the acidification stress by the algal host in the thallus-associated boundary layer due to photosynthesis in daytime. Despite the (presumed) biotic modulation by the host alga, we observed very severe effects on growth and settlement of the tubeworm *S. spirorbis* under the conditions of upwelling of year 2100. The calcifying and non-calcifying bryozoans *E. pilosa* and *A. hirsutum* were not affected by elevated pCO_2_ but rather favored under present-day upwelling conditions. This study shows for epibionts that (i) calcifiers may not all be “losers” in a high pCO_2_ ocean and (ii) small scale processes at the habitat or microhabitat scale could mitigate the impact of acidification on species and communities.
